# Perceptions of the determinants of health and cancer: trends in discourse and level of argumentation between girls and boys aged 6 to 18

**DOI:** 10.3389/fpubh.2025.1390084

**Published:** 2025-02-17

**Authors:** Maéliane Deyra, Chloé Gay, Laurent Gerbaud, Pauline Berland, Frank Pizon

**Affiliations:** Université Clermont Auvergne CNRS, SIGMA Clermont, Institut Pascal, Clermont-Ferrand, France

**Keywords:** perceptions, health determinants, children, gender, girls, health conceptions

## Abstract

**Aims:**

To characterize the discourse trends and level of argumentation of girls and boys by analysing the differences in the conceptions and systems of conceptions of children and adolescents aged between 6 and 18.

**Materials and methods:**

This is a multi-centre qualitative study in the human and social sciences, based on data collected in two different phases using two different tools: e.Photoexpression©, which deals with health in general, and Photonarration, which deals with cancer. The aim of this open, exploratory method, which uses photographs, is to gather data on the experiences and knowledge specific to each child and adolescent. The informative value of data from qualitative collection tools, combined with a mixed analysis methodology, enabled us to characterize the differences in perceptions of health determinants and cancer between girls and boys aged between 6 and 18.

**Results:**

4,174 productions were collected from 1,068 children aged 6 to 18, identifying 30 determinants of health and cancer. For all of these results, there were significant gender differences from a very early age: boys focused on leisure activities and physical activity, while girls took a more global view, focusing on the environment, food, emotional aspects of social relationships, hygiene, care, prevention, etc. As they got older, the focus shifted to health determinants. As we get older, we see a change in discourse trends and in the level of argumentation, with girls becoming richer and boys poorer.

**Discussion and outlook:**

The trends in girls’ and boys’ discourse on what, in their view, determines health demonstrate the interest and relevance of adapting the prevention methods used as closely as possible to the conceptions of children and adolescents. The differences observed between boys and girls are a crucial lever that takes into account the specific characteristics of a population. They offer the possibility of taking more effective action, both in the context of interventions aimed at teenagers and in support of decision-making in the context of prevention policies.

## 1 Introduction

Our living environments are becoming increasingly complex, and our societies are resolutely facing major health challenges (1). These contextual changes are once again bringing to the fore the importance of public health for young people, taking into account the specific characteristics of each individual (2, 3). The prerequisite for this is to attach importance to the concepts that enable us to understand how young people perceive the world around them (4, 5). Indeed, children’s and adolescents’ descriptions of health reveal avenues to be followed, as “neglecting people’s representations of health in health education, health promotion and planning programmes means depriving ourselves of the keys to understanding many factors of success or failure” (6). This point of vigilance is essential and fundamental if we are not to impose our adult vision, influenced by our own conceptions, on a young audience. Conceptions bring together what enables individuals to characterize their health and what determines it from a biopsychosocial perspective (7). Investigating health conceptions remains a complex process and a real challenge for research, even though life contexts are constantly changing (1–3). This leads us to reflect on the place we attribute to others, in their uniqueness and capacity to develop, and therefore to identify and better understand what we need to interact with in the context of prevention policies (5, 8).The idea is to start from what people know and teach them to understand how they make decisions about their health, so as to increase the number of alternatives available to them and when we talk about health, we need to identify what people understand about what can determine them. The determinants of health are the socio-economic factors that act interdependently with the environment and individual behavior, characterising health status through complex interactions (76, 9, 10). Today, it is mainly chronic diseases, including cancer, that affect health, diseases that are strongly correlated with living conditions and the environment (11). To promote health and reduce the risk of developing cancer, we need to act on the determinants of health which, apart from certain specific types of cancer, are similar to the determinants of cancer (12). In fact, the increase in cancers and the general state of health of the population depend on several similar factors: demographic growth, ageing and changes in the prevalence of certain causes of pathologies associated with social and economic conditions, poverty and infections (13), individual behavior and also environmental determinants (12, 14–16).

These different dimensions are found in the many models presenting the determinants of health (17–19), which tends to confirm that there is a conceptual mesh between the notions of cancer determinants and health determinants, which ultimately resemble and converge. Dahlgren and Whitehead’s 4-level rainbow model offers a new perspective on the issue of health determinants: the importance of behavioral determinants is reduced and socio-economic, cultural and environmental conditions, placed at the centre of the model, constitute the structural determinants (10).

Added to this is the question of health inequalities, which constitutes a new key to understanding health (9, 17, 20) and which opens up the cumulative dimension of the dangers weighing on health (21, 22): a child exposed to one risk factor could accumulate others and develop certain pathologies in adulthood (23). This idea introduces the life trajectory process, which sheds dynamic light on the relationship between health determinants. The logic of interlocking levels reflects the idea that health is an evolving construct that shapes the physical and psychological state of the individual. These theoretical elements form the basis of a general, scientific framework, but since 1991 (10), society has evolved, incorporating new dimensions into health issues. These models do not, therefore, meet all the requirements of adaptability to the context and to the greatest number of people (1–3).

This is why it is necessary to identify young people’s conceptions of the determinants of health in order to better understand how they interact and how we can intervene as effectively as possible in prevention by avoiding copying a model designed by adults for young people (24–26).

The initial results of the ‘Déterm’Ados’ research project, of which this study is a part, financed over 3 years by the Ligue nationale contre le cancer (2019–2022), revealed a strong polarization of the discourse of children and adolescents around individual determinants, minimising environmental determinants (26, 27). These data are of concern given that 40% of cancers are attributable to individual behavior and that for the majority of cancers, the causes are linked to environmental determinants on which there is little that can be done at an individual level (12, 16).

However, the discourse of children and adolescents remains mainly centred on diet, alcohol and tobacco consumption and physical activity (26–29). Can this be explained by behavior observed in adults or by prevention messages conveyed by society? Among adults, there are disparities: men are more physically active than women, regardless of the country to which they belong (30). To take the analysis a step further, it seems interesting to see whether conceptions of health already vary from childhood onwards, and whether there are gender differences and variations on these issues. The aim is therefore to characterize the significant differences between the conceptions and systems of conceptions of girls and boys on the determinants of health and cancer to see how they influence decision-making for health in adulthood.

## 2 Materials and methods

### 2.1 Study framework

This is a comprehensive qualitative study of the Human and Social Sciences carried out among children and adolescents from primary schools to high school in the Auvergne-Rhône-Alpes (AURA) region. The schools were located in a variety of contexts: rural, urban, Réseau d’Education Prioritaire (REP)^1^, small or large school groups, in order to provide a territorially diverse sample. A total of 12 schools with different characteristics from the Clermont-Ferrand Académie and Rectorat were selected on a voluntary basis. All age groups (from 6 to 18) and school levels were surveyed.

### 2.2 Data collection

The data was collected using a multi-phase qualitative study protocol based on two tools: e.Photoexpression©^2^ and Photonarration.

The first phase with the e.Photoexpression© tool consisted of asking the children and teenagers to choose two images from 40 photographs that best answered the question: ‘Choose an image that you think represents good health’ and ‘Choose an image that you think represents bad health’. Once the selection has been made, each person explains in writing the reasons for their choice. Composed of photographs representing the biopsychosocial register (medicines, a smile, the family, etc.) as well as more neutral photographs (a flower, a landscape, etc.), e.Photoexpression© allows freedom in what it shows, offering a very wide range of ideas on the determinants that have a positive or negative influence on health. This tool, validated and referenced by PIPSA, registered with INPI, is protected by copyright (7). Several criteria were taken into account when compiling this body of photographs. Firstly, an aesthetic criterion including sharpness and framing. Then there was a criterion of meaning, which corresponds to the senses emanating from the image. The image must be open to offer a diversity of interpretations. Finally, a criterion of homogeneity that translates into a fairly broad spectrum of photographs allowing everyone to express themselves.

Photography is a lever for expression, enabling us to put into words what we see in the image. What is conveyed by the photograph contributes to the emergence of language, from a basic level of simply describing what is present in the photograph to a much more elaborate level of argumentation linking several dimensions. Vygotski (31) work has shown that, through language, children gradually organize and develop their thinking. Mediation through images therefore creates a space that frees up speech while respecting essential ethical considerations. It gives children and teenagers the opportunity to structure their thinking and highlight the quality of what they say. Through images, they find their own way of talking about health and cancer, in their own words. They then become actors in the collection and provide an argued and personal interpretation of the photographs they have chosen (32). The second phase, using the Photonarration tool, consists of choosing an unlimited number of images from a collection of magazines, cutting them out, assembling them and pasting them onto an A3 sheet to respond to the following instruction: ‘create an assembly of images from the magazines provided that shows the causes of and protective factors against cancer’. Each image was accompanied by a text on the back of the sheet answering the following questions: ‘What, in the images you have chosen, represents for you what causes cancer?’ and ‘What, in the photographs you have chosen, represents what helps to prevent cancer?’ The main eligibility criterion for magazines offered to children is diversity (n > 100 copies) in terms of themes (sport, nature, hobbies, decoration, themed magazines, health, etc.), targeting different ages (children, adults, senior citizens) and potentially addressing all the determinants of health. The corpus is enriched by leaflets from supermarkets (food, leisure, other consumer products, etc.). The aim of Photonarration is to increase the density of the discourse and the emergence of an argument that will give a better understanding of how the concepts fit together. The narrative dimensions of this tool encourage a better understanding of the process by which children link conceptions relating to different areas such as sport, food, the family, interpersonal relationships, emotions and feelings with the theme on which they are being questioned (health in general and cancer).

### 2.3 Ethical considerations

This open and exploratory methodology, using photographs, is a way of encouraging people to speak out, and enables us to gather information on how each child and adolescent views the determinants of health and cancer (27). These image-mediated media play an essential ethical role, ensuring that there is a distance between the topic being discussed and the young person. Young people do not talk about their personal situation, but only about the subject of this study: the determinants of health and cancer.

Each participant was given a code which made it easier to trace what was said at each stage of the data collection. The absence of nominative data also guarantees that the children cannot be identified and remains anonymous. This research in the Human and Social Sciences is not ‘research involving the human person’ (RIPH). The Déterm’Ados protocol was granted ethical clearance outside the scope of the Jardé Law by the Comité de Protection des Personnes (CPP) Sud-Est VI, with a view to publishing the results. This intervention protocol was designed on the basis of data from the literature, international ‘Good Clinical Practice’ (GCP) recommendations from the Declaration of Helsinki, hypotheses from the researchers’ professional experience and the methodology used in previous health and addiction studies (5).

The children and their parents were also informed, in comprehensible terms based on an ‘Easy to Read and Understand’ (FALC) approach, of the entire process, from the objectives of the study to the nature of the information collected, as well as of their right to withdraw at any time and to refuse the use of the data collected. The session could be interrupted at the request of the child, who was free to leave the room early if he or she so wished, as participation was voluntary.

The involvement of young people in research is essential to guarantee their right to take part in the debate on issues that affect them (International Convention on the Rights of the Child) and to improve the value and validity of the results. It ensures that their experiences and perspectives are properly recorded, providing accurate and specific information, respecting their voice. Children and teenagers were treated equally and without exclusion. To achieve this, and to respect the abilities of each individual, the conditions under which the data was collected were adapted. The youngest children and all those for whom writing is still fragile were accompanied by researchers or teachers in the form of dictation to an adult, who transcribed only the child’s words, without helping or influencing them. This approach helps to reduce inequalities in writing skills.

### 2.4 Data analysis

This qualitative data collection made it possible to stabilize a mixed analysis protocol, which was necessary to produce in-depth results. The data collected using e.Photoexpression© and Photonarration were analyzed quantitatively and qualitatively by a multidisciplinary team in order to group them into blocks of meaning structured around key words based on its previous work categorising health verbatims according to the biopsychosocial model (5, 77). The aim was to identify references to determinants of health and cancer in the discourse of children and adolescents and to characterize the differences between the conceptions and systems of conceptions of girls and boys. A content analysis was conducted using the bottom-up/top-down categorization method and the ‘heap’ procedure (78, 79). This made it possible to categorize the verbatims referring to a health theme, emotional dimensions, hygiene, care, protection, relationship with the environment, factors of personal fulfilment and development, social relationships, health recommendations and support from the social sphere, or the notion of health capital. In order to check the accuracy of the nesting of the different levels, these categories were stabilized on the basis of the children’s discourse, and a back-check was made with the health determinants model, the theoretical foundation of this study (10).

In addition, in order to limit indexing bias, the data was encoded three times, ascending and descending by each researcher, which made it possible to check that the domains, rooted in the determinants of health, grouped the categories together. Exchanges between researchers from several disciplines contributed́ to enriching the analysis and reinforcing the reliability of the results through a cross-view and different expertise. To avoid any over-interpretation of the verbatims collected, where there was any doubt about indexing them, contentious data was discarded after a thorough examination. The triple encoding of the qualitative analyses then enabled descriptive statistical analyses, enriched by regular feedback on the verbatims highlighted during the qualitative analysis. The quantitative descriptive analysis, carried out using SAS© version 9.40 software, revealed the general trends in the study, presented in the form of frequencies and recurrences. All statistical tests were carried out with a risk of error of the first kind *α* of 5%. A multiple correspondence analysis (MCA) was carried out to study the organization of the designs and test the hypotheses identified. We also sought to identify context effects for the frequent conceptions by studying the links between these conceptions, age, sex, class and type of school, using logistic regressions.

These exchanges between the qualitative and quantitative analyses made it possible to carry out adjustments and retrocontrols in order to maintain the authenticity of the data, to preserve their level of granularity to avoid over-interpretation (33). Finally, the Gephi 9.2 data visualization software was used to position the relationships established between the health determinants perceived by the children and adolescents by means of a pictorial and synthetic representation of the systemic links established between the conceptions.

To select the children whose systems of conceptions were to be analyzed at individual level, we retained the main trends revealed by the statistical analysis, which we compared with the children’s and adolescents’ verbatims. This makes it possible to keep the dominant discourse with the overall quantitative data, while retaining the original meaning. This approach thus offers a new phase of qualitative feedback with image modelling of children’s and teenagers’ conceptions of health, which enables us to retain the authenticity of the source data. By analysing the global, i.e., the collective, and then the specific, i.e., the individual, we move from a mapping of conceptions to a pictorial representation of conception systems.

## 3 Results

The corpus of this study is based on 4,174 productions (4 productions per child), collected from 1,068 children and teenagers aged between 6 and 18 (47% girls and 53% boys) in five elementary schools, four collèges and three lycées. These schools, in the Allier and Puy-de-Dôme départements, were recruited on a voluntary basis and were of different backgrounds: rural, urban, Priority Education Network (REP) or not, vocational or general stream, small or large school group. During the first phase of data collection, 1,059 children and teenagers took part in e.Photoexpression© and produced 2,118 works of art. In the second phase, 1,028 were interviewed and 2056 Photonarration productions were collected. The difference in the total number of participants between the two phases is explained by the absence of children due to minor illnesses (gastro-enteritis, rhinopharyngitis, angina), personal reasons or transport difficulties due to the snow.

We will present the results along two main lines: changes in the discourse patterns of girls and boys, broken down by age group, and changes in the level of argumentation and the systemic view that girls and boys have of the determinants of health and cancer, again broken down by age group.

### 3.1 Changes in the speech patterns of girls and boys aged 6 to 18

#### 3.1.1 Speech patterns in children aged 6 to 11

The first results of this study have been published (25, 26, 34). and highlight two major trends in children: the under-representation of environmental determinants and the over-representation of individual determinants. They remain centred on rationales that favor biological aspects and minimize dimensions linked to the living environment in the broad sense. In view of our objectives, we are interested here more specifically in the trends in discourse revealed as a function of gender.

**Figure 1:**
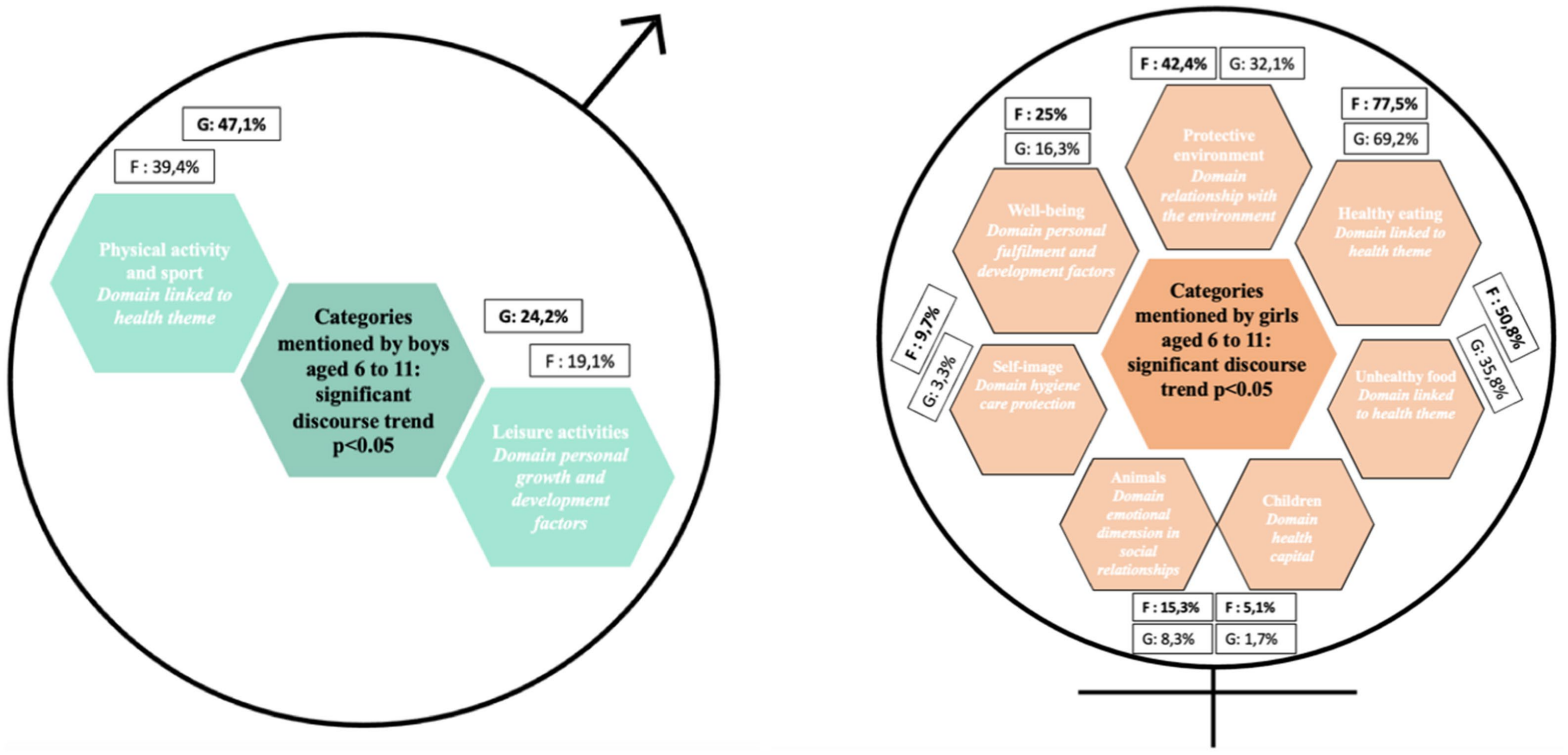
Categories mentioned by boys and girls aged 6 to 11 for e.Photoexpression and Photonarration: significant speech trend *p* < 0.05.

**Figure 2:**
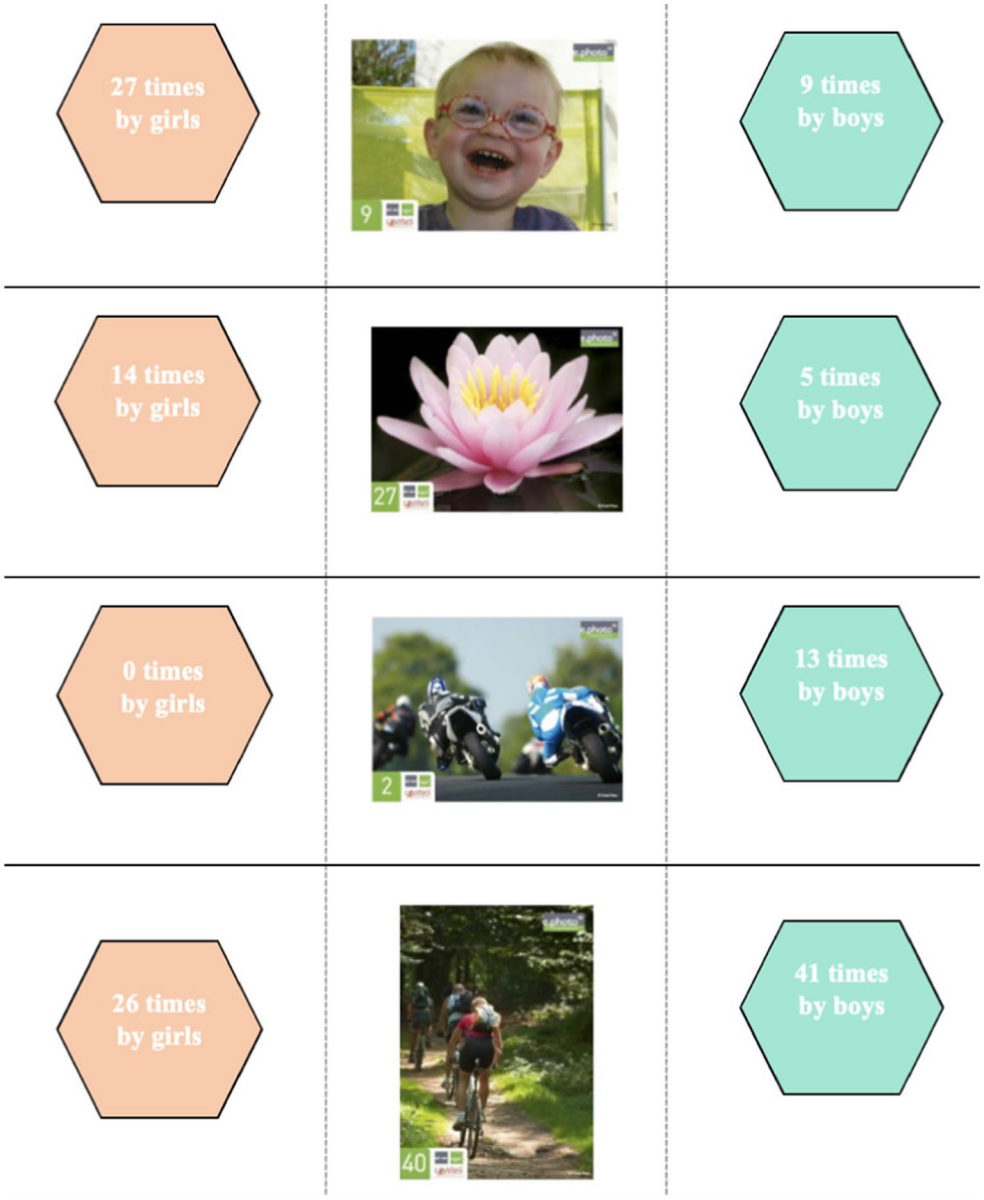
Dominant choice of photographs according to gender and speech patterns among 6–11 year-olds.

**Figure 3:**
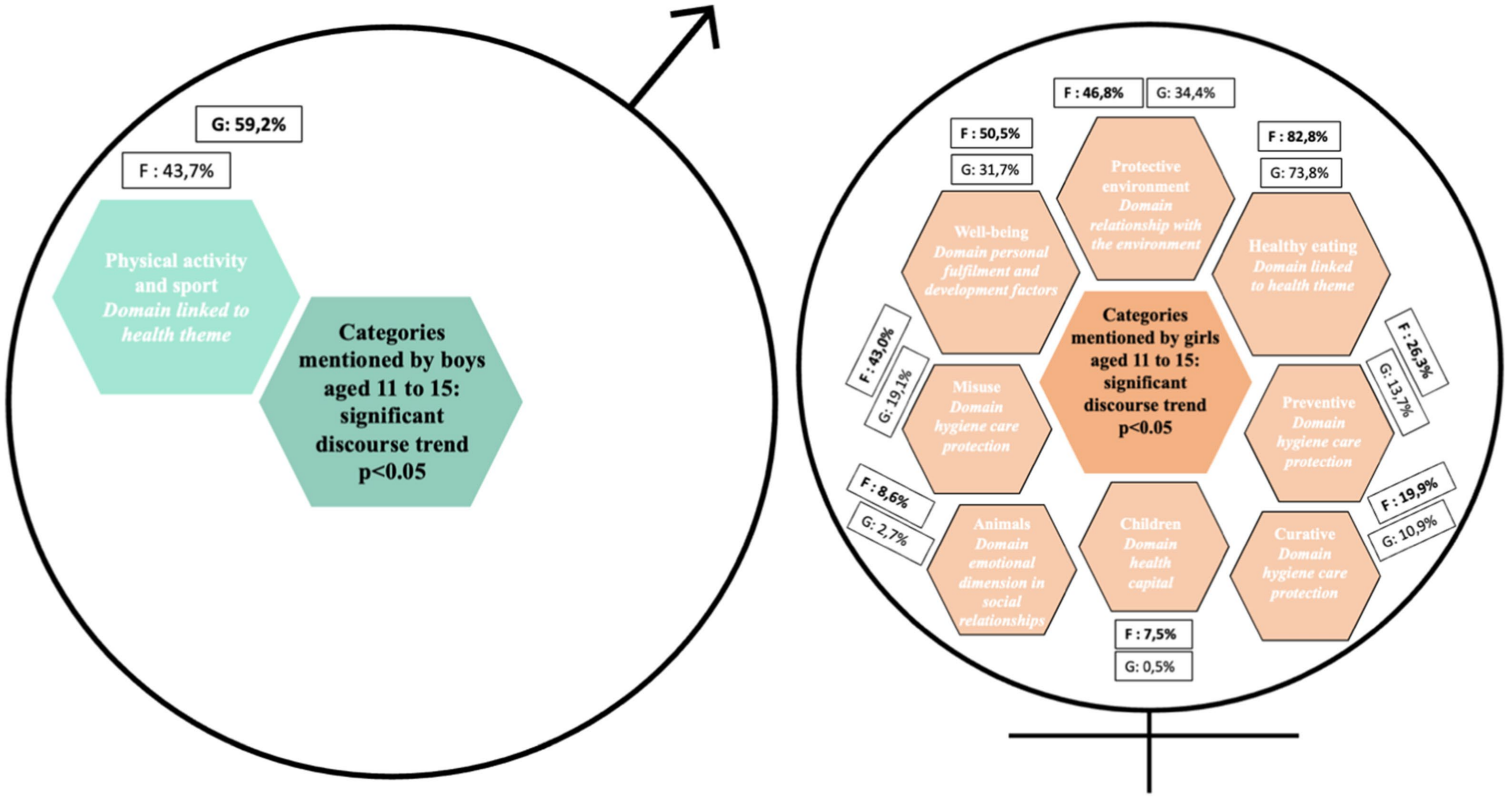
Categories mentioned by boys and girls aged 11 to 15 for e.Photoexpression and Photonarration: significant speech trend *p* < 0.05.

In both phases of data collection, boys were more likely than girls to mention physical activity, sport and leisure activities as criteria for good health or factors that protect against cancer (Figure 1). Conversely, girls were more likely than boys to talk about the environment, diet, the emotional dimension of social relationships (Figure 2), and hygiene, care and protection (Figure 3). In all, 12 determinants of health and cancer were mentioned significantly (*p* < 0.05) by girls aged 6 to 11, compared with 2 by boys (28).

**Figure 4:**
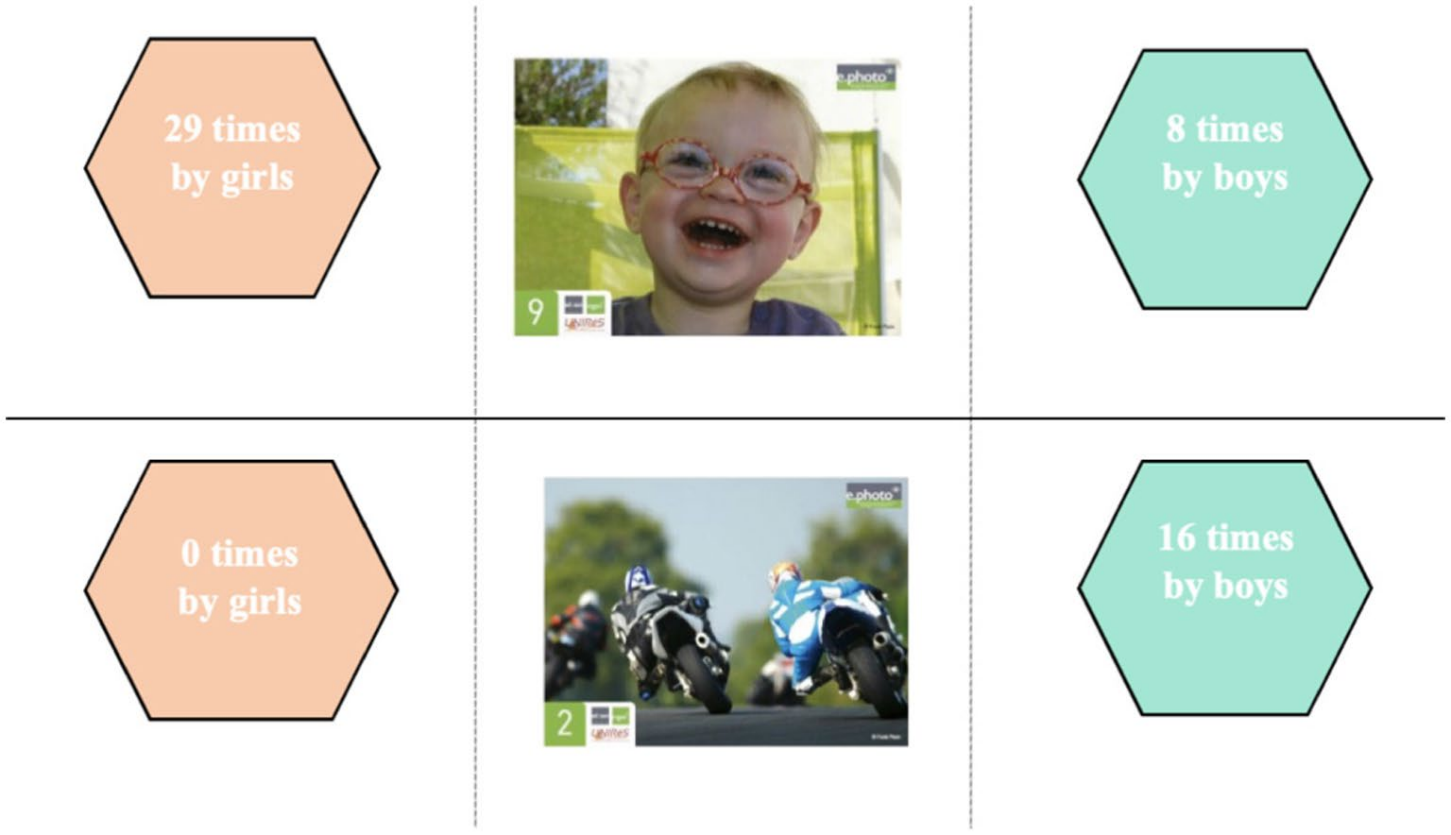
Dominant choice of photographs according to gender and discourse trends in the 11–15 age group.

The choice of photographs taken by the children during the e.Photoexpression© (first phase) confirms these gender-based discourse trends. In order to identify differences in the choice of photographs, a standard deviation threshold was defined between the number of times an image was chosen by girls and the number of times the same image was chosen by boys. This threshold was set at a minimum of 10 (Figure 4).

Although photograph 40 was chosen by both girls and boys, it was the boys who mainly identified it as a way of talking about physical activity and sport: “sport helps you develop your health” (AMCE220M); “you have to exercise” (AMCM214M); “you do sport and sport is good for your health. Our bodies actually like it. We sweat and everything, so it rejects germs and everything” (GUCE14M). The girls, on the other hand, chose it to talk about sport but also for the environmental aspect to which it also refers: “it helps us breathe in the trees around us” (LECM1D12F); “because they do sport and it’s good for our health and we breathe better in nature” (AMCM124F) or again in relation to social links “because it’s good for our health to cycle as a family” (MMCM17F).

Photograph 2, which echoes leisure activities, was selected exclusively by boys: “because it’s sunny and it feels good to ride a motorbike” (AYCE210M); “for me, riding a motorbike is good for your health” (GUCP4M); “riding a motorbike is good for your health, it’s a hobby I enjoy” (MMCE110M).

Photograph 9 aroused most interest among the girls, who associated it with happiness and joy: “He′s happy, he’s laughing, he looks healthy” (AMCM219F); “I took it because he’s happy and when you are happy you are healthy” (AYCE1R3F); “It’s nice to smile, you are happy so you are healthy” (AYCE212F); “because he’s happy and that makes me think he’s having fun so he’s not sick” (AYCE226F); “he’s very happy which means he feels love inside” (GUCE110F). The few boys who chose it gave the same reasons, but some also emphasized the fact that they were having fun, i.e., playing, which links their discourse to the “leisure activities” theme: “the boy is having fun, he’s in good health” (AYCPV19M).

Photograph 27 was mainly identified by the girls. Some chose it because it represents good health in an imaginative way: “it looks healthy because its petals are not damaged and are well spread out” (AYCE1R6F); “because this flower is open, and when it is open it is healthy” (AYCE1R5F). The boys also chose it for these reasons: “the flower is healthy because the flower is always alive” (AMCE223M); “because there are no germs” (GUCE25M) or in a purely descriptive way: “because it is pink and yellow” (LECE115M); “because it smells good” (GUCP1M). In the same descriptive vein, some girls emphasized the beauty of the photograph: “I think this picture is happy and healthy because it’s a pretty pink water lily with a yellow centre, these are warm colours and it’s on water” (AMCM110F). These comments reveal the symbolic importance attached to the image by the girls. Most of them also chose it to talk about nature and the importance of protecting the environment: “we have to take care of nature to reduce pollution” (LECM1D10F); “flowers are important for nature” (AYCE1R13F). Others mentioned the role they play in our health: “Flowers protect nature and take care of people. It makes me think of yoga and festivities” (LECE111F); “I chose it because flowers help you breathe” (AYCPV7F); “because we need nature to live and we need trees to breathe” (AMCM122F); “we need flowers for the bees to gather pollen, to make honey, and honey can heal and is good for your health” (GUCP18F). For a girl in CP class, this photograph echoes the emotional dimension and the social bond: “the flower looks like love, we feel good, we do activities together, we are happy” (GUCP2F).

#### 3.1.2 Speech patterns in adolescents aged 11 to 15

**Figure 5:**
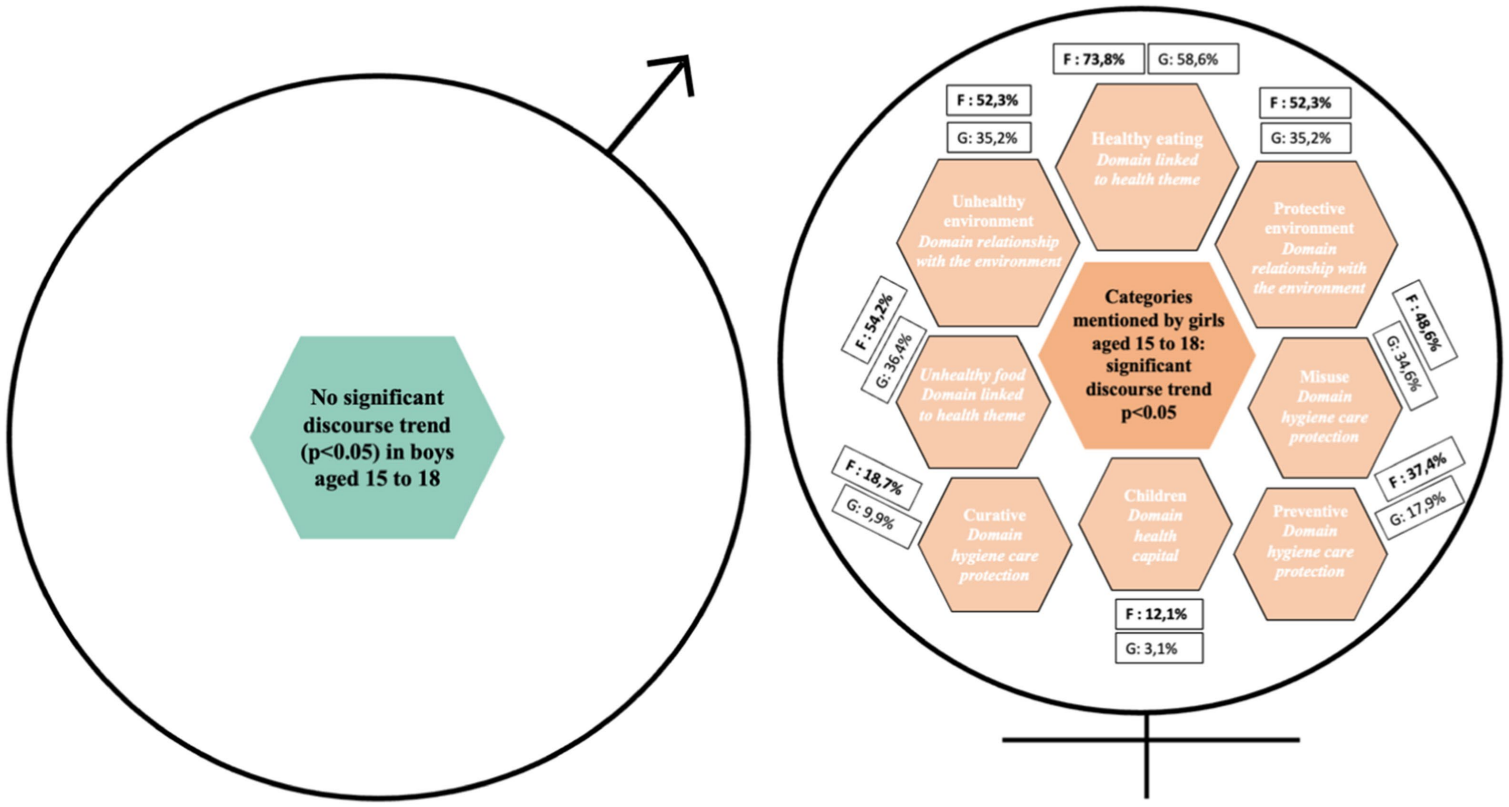
Categories mentioned by boys and girls aged 15 to 18 for e.Photoexpression and Photonarration: significant speech trend *p* < 0.05.

**Figure 6:**
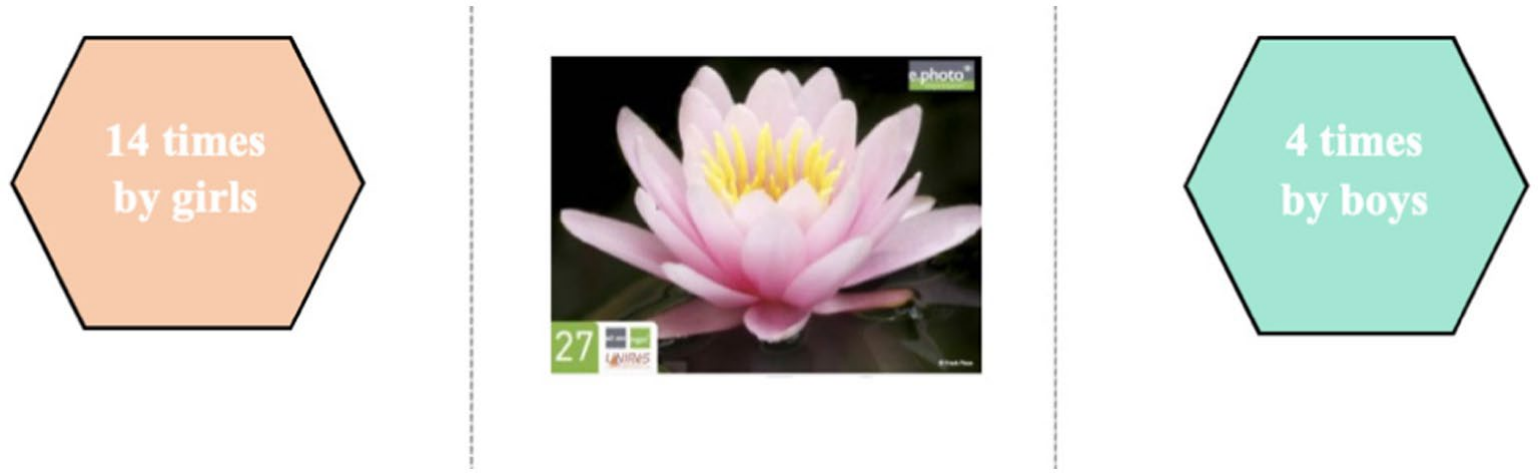
Dominant choice of photographs according to gender and discourse trends among 15–18 year olds.

**Figure 7:**
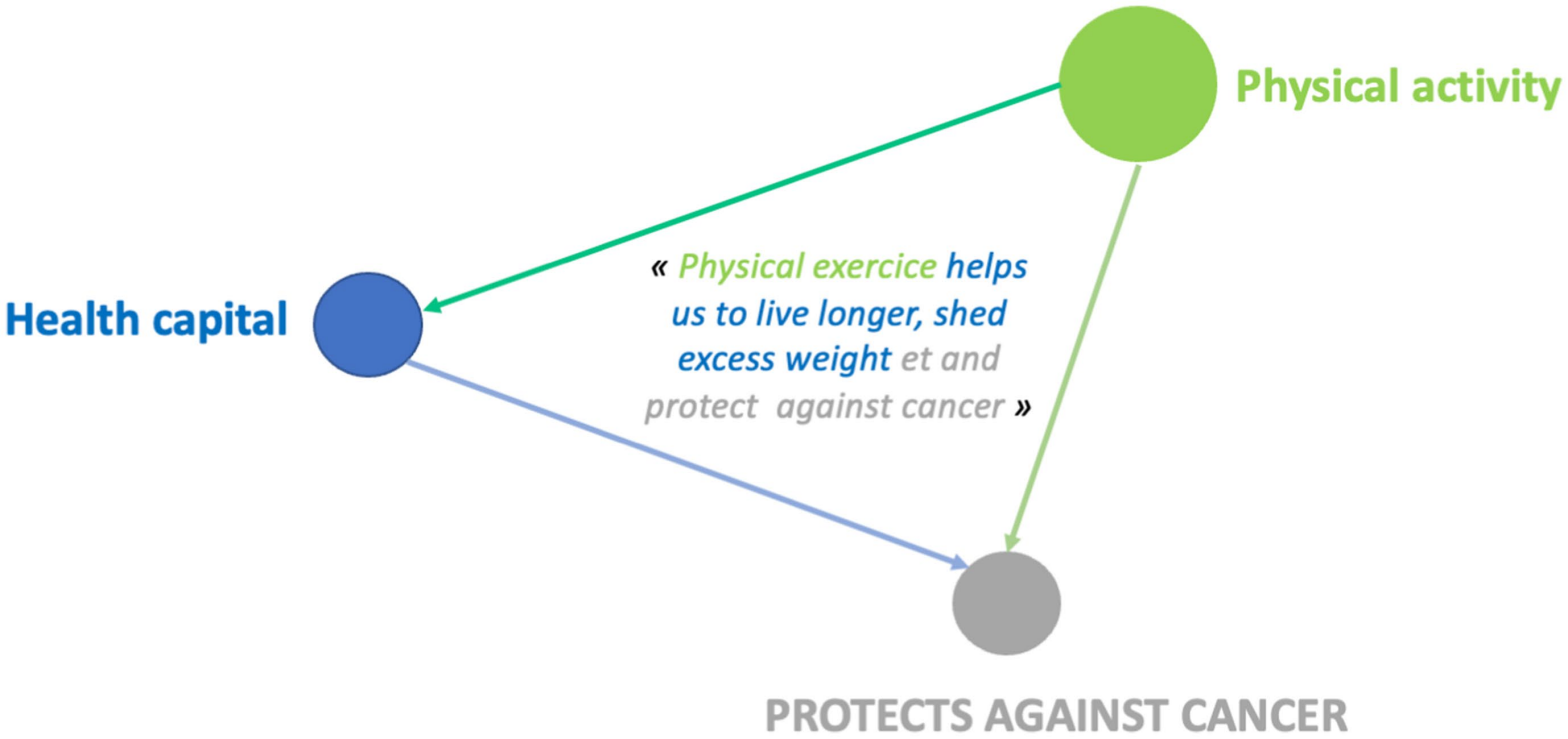
Example of design systems for the “physical activity” category for 9-year-old boys.

In both phases of data collection, boys were more likely than girls to mention physical activity and sport as a criterion of good health or as a protective factor against cancer (Figure 5). Conversely, girls were more likely than boys to talk about the environment (Figure 6), diet, the emotional dimension of social relationships, and hygiene, care and protection (Figure 7). In all, 11 determinants of health and cancer were mentioned significantly (*p* < 0.05) by girls aged 11–15, compared with 1 by boys (28).

The photographic choices made by the 11–15 year-olds during the e.Photoexpression© (first phase) confirm these trends in discourse according to gender.

Although photo 9 was chosen by boys, there was a predominance of girls, similar to that identified among the 6–11 year olds. They also associated it with joy and being happy: “joy is important for living because often when you are ill you are sad” (LP6E16F); “I think the child is happy, he’s funny, he’s enthusiastic” (LP5E6F); “I made this choice because for me being healthy starts with being happy” (LP4E8F).

The 11–15 year-olds linked their self-image to the notion of happiness: “because you can see that he’s smiling, that he’s happy and that he’s taking responsibility for himself” (LP6E1F) and identified well-being as a resource: “smiling feels good, you feel better. Even if you have problems, smiling helps you overcome them, it makes things easier” (FV3E19F). The few boys who chose it gave the same reasons: “I chose this image because the child is smiling” (AB3E12M); “No. 9 because children mean good humor, and therefore good health” (LP3E10M), but some associated it with doing something they enjoy, which goes back to the “leisure activities” theme, predominant in their discourse: “I chose this image because, for me, if a child is laughing, it means they are fulfilling themselves in the activity they are doing or that they are in good health” (LP5E1M).

Photograph 2 was only selected by boys, as was the case with the 6–11 year olds. Although some of them also mentioned “leisure activities,” as did the younger ones, the 11–15 year-olds associated it more with physical activity and sport: “because motorcyclists are top-level sportsmen and women, and a great sportsman or woman must be in good health” (LP3E18M); “because motorcycling is a sport because you are always on the move, so it works the body, especially the upper body” (EG4E18M). For them, sport limits health problems and you also need to be in good shape to do it: “I chose number 2 because motorcycling involves physical activity, which limits cardiovascular risk” (FV5E14M); “I chose this image because to be a motorbike racer, you need to be the right build and not have any health problems” (LP5E4M).

#### 3.1.3 Speech patterns in adolescents aged 15 to 18

Among 15–18 year-olds, girls have a more global view of the determinants of health and cancer than boys. They attach importance to diet, the environment, hygiene, care, prevention and health capital (28). In all, 13 determinants of health and cancer were mentioned significantly (*p* < 0.05) by girls aged 11 to 15. In contrast to the 6–11 and 11–15 year-olds, no dimension was mentioned significantly in the discourse of 15–18 year-old boys compared to that of girls (28).

The choice of photographs in the e.Photoexpression© (first phase) confirms this result.

There is no predominant choice of photograph for boys. As for the girls, n°27 was again chosen, as it was for the younger age groups. The idea of a protective environment was still present in their comments, but the photo was chosen more for its pictorial aspect, representing good health: “Nature is the most natural thing there is when it has not been damaged by humans. This flower is beautiful and well developed, so we can say it’s in good health” (CHERE29F); “the flower is colourful and beautiful, it represents good health. What’s more, it has not wilted, so it’s healthy. A flower generally represents life, happiness and therefore also good health because it is ephemeral” (JANDE53F). We can see that the symbolic significance given to the image by the girls persists as they get older.

The girls associate psycho-affective dimensions with this photo: “good health: the image of the flower, for me it means purity, life, freedom” (CHTERP3F); “this image evokes good health for me because you see the flower open it evokes positive thoughts it shows a clean environment” (CHEREP7F); “the lotus is a flower symbolising serenity and purity. It is also a yoga position, a practice that promotes well-being and helps us to refocus on ourselves. The purity of the lotus is similar to the purity of good health and a healthy diet. It’s a plant that shows the importance of eating legumes, that goes with the flow, that shows that we need to be physically active, not necessarily intensely, but enduringly, and the importance of hydration” (JANDE22F). They also integrate biological dimensions by citing the healing role of nature: “it’s a flower and I find that it’s synonymous with good health, plus it’s very colourful and bright, it inspires naturalness and, for me, health. Also, some medicines are based on flowers or plants, which help to restore health” (JANDE21F).

We can see that, overall, the trends in the boys’ discourse correspond to protective determinants that are favorable to health, since most of them talk about physical activity and leisure activities that contribute to good health and a sense of well-being. The trends in girls’ discourse are generally favorable, but can also have an unfavorable impact. Diet, the environment and mental health are all factors that can have both a positive and a negative impact on health, or a protective or risk factor in the development of cancer.

The e.Photoexpression© tool has therefore revealed certain discourse trends, focusing on certain determinants, in particular individual determinants. The choice of images can also influence this result. It is therefore interesting to look at the data collected during Photonarration, which allows a wider choice of images and a broadening of conceptions (27).

### 3.2 Evolution of the level of systemic argumentation between boys and girls aged 6 to 18

Having mapped conceptions on a collective scale, we then turned to systems of conceptions on an individual scale to identify the way in which the conceptions of a single child or adolescent are connected and interrelated. The aim of this approach is to highlight the existence of not one but several conceptions which, from the child’s or adolescent’s point of view, enable them to respond to the life situations they encounter. We seek to highlight the causal links (or lack of them) present in his or her discourse. This second phase also reveals other results based on gender. When we look at the discourse collected via Photonarration to see whether the boys and girls we met establish links between the determinants they identify, we see that overall, the boys present what they believe helps protect them from cancer or what can lead to it by listing the determinants, with very few links between them. The girls, on the other hand, mention a correlation between the various factors they cite in their arguments.

We will now focus on the categories that are significantly more common in boys than in girls, in order to identify the links established between these categories and one or more other determinants, as a function of gender and age.

#### 3.2.1 Levels of systemic argumentation between girls and boys in the 6–11 age group

When asked what helps protect against cancer, 10 boys mentioned physical activity and sport, linking it to just one other health determinant, compared with 16 girls who linked it to two or more other determinants.

For leisure activities, 7 boys linked them to another category compared to 14 girls who linked them to at least two other determinants.

The links between health determinants made by boys reflect simple design systems, associating two determinants with each other. Girls, on the other hand, develop connections with more determinants, which then constitute more complex conceptual systems. They have a more systemic and global vision of what they believe determines health.

#### 3.2.2 Levels of systemic argumentation between girls and boys in the 11–15 age group

When talking about what helps to protect against cancer, boys in this age group also tend to cite different determinants in succession without linking them together, even when these are dimensions that are predominant in their discourse (physical activity and sport): “Fruit and vegetables are good for your health. Playing sport is good for your health” (LP6E8M).

22 boys mentioned “physical activity and sport,” linking it to another health determinant, compared to 54 girls who linked it to two or more other determinants.

#### 3.2.3 Levels of systemic argumentation between girls and boys in the 15–18 age group

**Figure 8:**
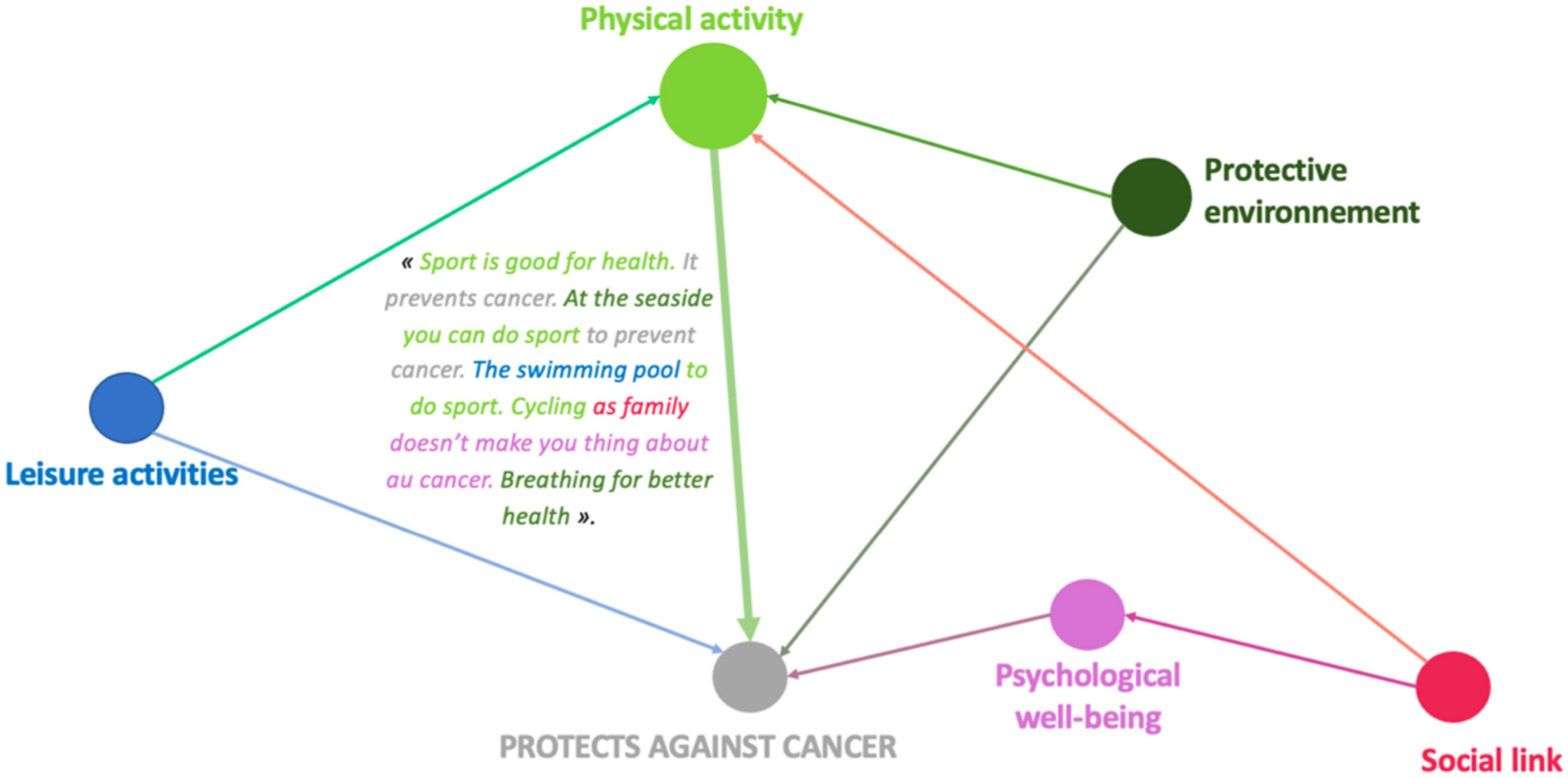
Example of design systems for the “physical activity” category 10-year-old girl (GUCM14F).

**Figure 9:**
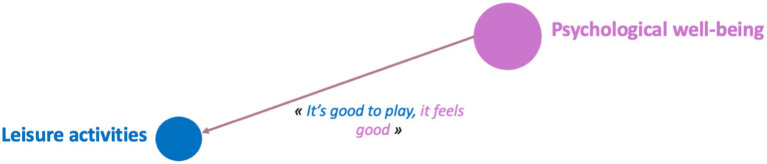
Example of design systems for the 8-year-old boy’s leisure activity category (MMCE17M).

As in the younger age groups, girls aged 15–18 attach significantly more importance than boys to several determinants (Figures 8, 9). Boys, on the other hand, do not have any dominant discourse compared to girls and unlike boys aged 6–11 and 11–15.

These results highlight the fact that the level of argumentation is higher among girls and that they have a more systemic view of health than boys, even when it comes to dimensions that they mention significantly more than girls. For this age group, we are therefore going to look at the use of psychotropic drugs, a theme to which the 15–18 year olds mostly refer, without distinction according to gender.

When talking about ill health and/or cancer, 73.8% of girls and 74.7% of boys mention tobacco, alcohol and drugs.

Given that they were mentioned almost equally, it would be interesting to see whether the level of argument used by girls is again higher than that used by boys.

Of all the young people interviewed, 24 girls and 12 boys linked the use of psychotropic drugs to between two and five factors. The boys rarely associated it with more than two factors. It was often the girls who mentioned more than 3 dimensions in addition to psychotropic drug use.

**Figure 10:**
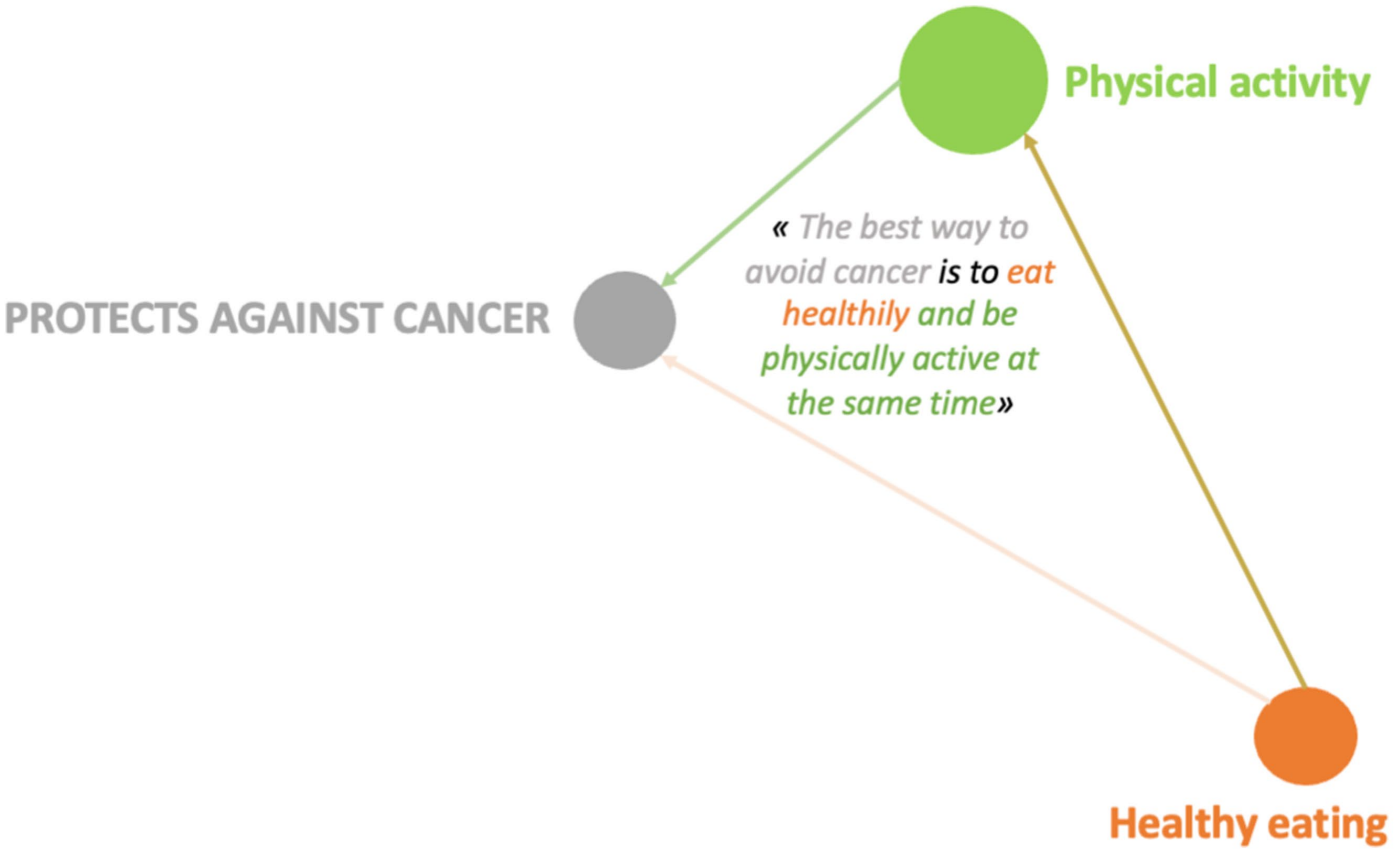
Example of design systems for the ‘use of psychotropic drugs’ category, 16-year-old girl (JANDE49F).

This representation image (Figure 10) meets a variety of dimensions that allow for consumption of psychotropes identified by people and people who are susceptible to cancer. Because this is the case, the full environment is similar to the tabac: “Totally that is used with the chemistry and artifice does not fit into the body without cigarettes.” The mal-être is also linked to the consommation of psychotropes: “The solitude of the mal is and is on this day, on the fumer or the air.” The ability to use the vaccine and prevention is shown on the depths of the dimensions that appear on the corners of the tabac and the alcohol: “The vision is the same sign of cancer […], this is important in this context, soon on a date and in your life.”

This design system reveals a lower poverty of connections between several determinants, characteristic of the expression collected among boys to define what can lead to cancer. As a reminder, only 12 of them associated the consumption of psychotropic drugs with other dimensions. This example confirms this result. This 17-year-old boy, taken as an example, connects only 2 determinants to the consumption of psychotropic drugs: the harmful environment and unhealthy diet.

**Figure 11:**
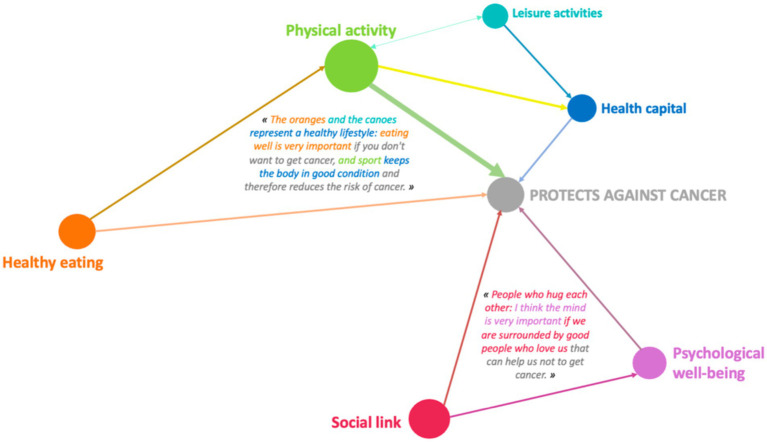
Example of design systems in the “leisure activity” category for 10-year-old girls (MMCM11F).

**Figure 12:**
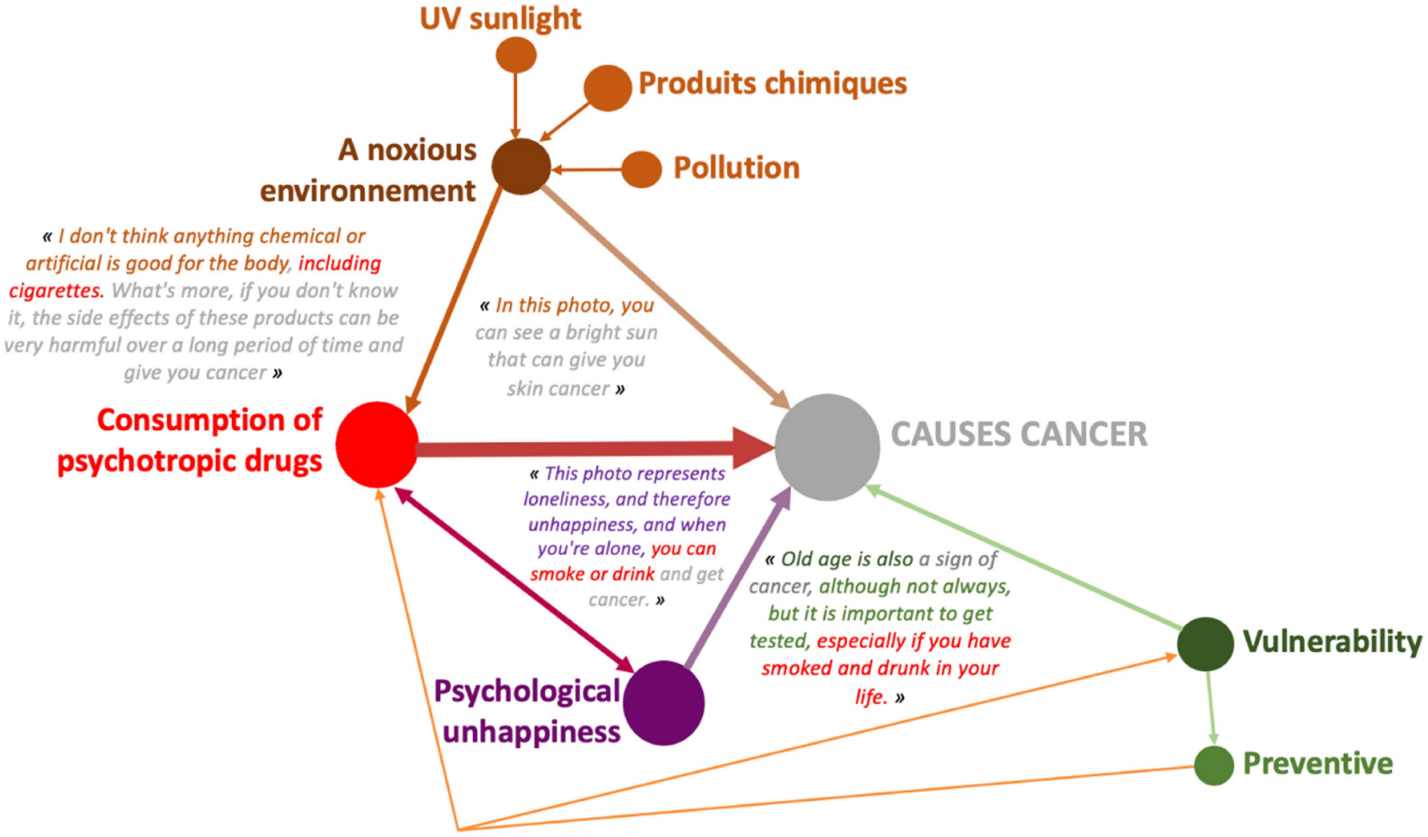
Example of design systems for the physical activity category for 14-year-old boys (LP4E13M).

**Figure 13:**
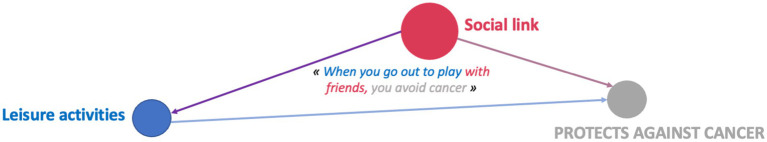
Example of design systems in the “leisure activity” category for 15-year-old girls (LP3E8F).

**Figure 14:**
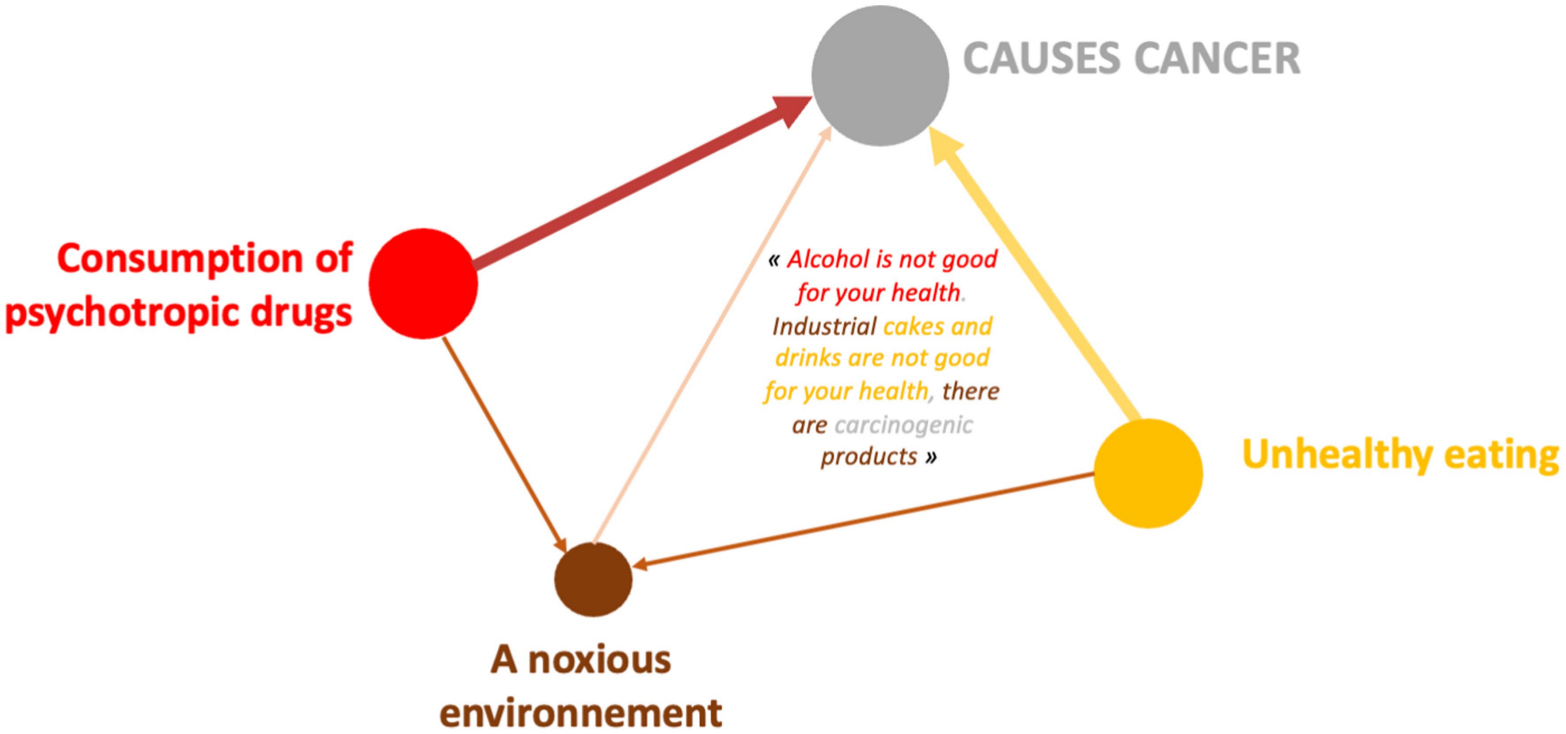
Example of design systems category “consumption of psychotropic drugs” boy 17 years old (GENDEP11M).

These pictorial representations allow us to realize that, whatever the age, girls have a higher level of argumentation than boys even when it comes to dimensions mentioned in a similar way by girls and boys. We find this observation in the case where the determinants are significantly more present in the speech of boys compared to that of girls. The pictorial representations of conception systems (Figures 7–14) indeed reveal more complex links among girls who consequently have a more global and systemic vision of what determines health.

## 4 Discussion

Using e.Photoexpression© and Photonarration, 4,174 qualitative productions were collected from 1,068 children and adolescents aged between 6 and 18. These productions highlighted significant differences between girls‘and boys’ conceptions of the determinants of health and cancer (28). We note:

- Boys focused on leisure activities and physical activity.
- Girls take a more global view of the environment, food, social ties and the family, well-being and psycho-emotional harm, as well as hygiene, care, prevention, and so on.
- The boys made little connection between the various factors they mentioned, with a moderate level of discourse,
- A strong link between the dimensions mentioned by girls, with a high level of argumentation, more commonly expressed symbolically.

Girls therefore have a more global and systemic vision than boys, and this difference intensifies as they get older. As they grow older, girls perceive health as a whole, integrating various dimensions to talk about what can constitute a health risk and what can help protect against it. These concepts give them a holistic view of health from an early age. This vision, which develops and enriches over the years, becomes poorer for boys. While girls see an increase in the number of health determinants cited, boys see a decrease.

The strengths of this study lie in the richness of the results presented. We know that understanding children’s and adolescents’ conceptions of health and more particularly of cancer is a relatively understudied research topić internationally (25). We can hypothesize that the issue of cancer retains an anxiety-provoking character in the social sphere that may explain why this area of research is poorly documented among children and adolescents in the general population. However, our results highlight the fact that they have many notions about health and cancer, which they express with ease when the conditions for collecting data are mediated by images (27). Their words show that they have resources within them that adults do not necessarily give them. The methodologies used here also make it possible, thanks to their complementary nature, to free children’s words and gain access to this surprising part of their conceptions (27). They offer the opportunity to a heterogeneous panel of children and adolescents (age and social context) to express themselves freely, to verbalize, in other words to put into words what, in their view, determines health. Taking an interest in what girls and boys have to say is a subject that has been very little investigated from a qualitative point of view, and that is what makes this study so special. Analysing these perceptions will give us a better understanding of their behavior and, as a result, enable us to provide them with support that is more tailored to their needs.

A limitation could be identified in the analysis of the data, and more specifically in the researcher’s interpretation of what the children and adolescents said. Nevertheless, the analysis protocol was designed to limit this bias. The data were subjected to a triple encoding process previously described in the methodology in order to avoid over-interpretation of the verbatim collected, and in the event of any doubt regarding their understanding, the research team evinced́ the contentious data by a meticulous examination of back and forth on the writings that made it possible to obtain additional indications. In addition, the approach taken is based on validated theoretical models recognized in the international literature (20, 35). Above all, it is based on a detailed and precise content analysis that respects the words of the children and adolescents, remaining faithful to the views of the girls and boys interviewed.

The question of gender has always been omnipresent in thinking about and organising the social world (36–38). Highly stereotyped generalizations about gender differences are not necessarily sectarian or false; they are based on social and historical reality (39, 40). They are often used to explain people’s behavior and conduct in terms of who they are: women, men, children, adults, young people, old people, etc. (41, 42). If we look at the theory of social roles (43), we see that men and women are inclined to have different expectations, expectations which they conform to and which legitimize asymmetrical social situations where the balance of power is more or less one-sided (44). The social roles assigned to women encourage them to behave in an expressive and emotional way, whereas the roles assigned to men lead them to focus ‘in an instrumental and non-emotional direction’ (45). These theories are interesting to compare with the results of our study. The girls we met, aged between 6 and 18, were predominantly concerned with psycho-affective well-being and psycho-affective harm, whereas the boys said very little about these issues. An analytical look at the photographs chosen by the girls shows that there is a significant pictorial dimension to the discourse, revealing a more advanced level of conceptualization than that of the boys. The verbatims associated with these photographs demonstrate the symbolic significance attached to images by girls from an early age. And this assimilation of symbolic language, like the systemic and complex vision they have of health, is maintained and confirmed as they get older. Girls talk about health and well-being by establishing more links with emotional dimensions, going beyond simple health behaviors (46–48). Our findings complement this observation with metaphorical, representative and allegorical dimensions. This way of thinking echoes Jacques Fortin’s work on the health paradigms underlying health education interventions, and more specifically the so-called ‘ecological’ paradigm, which focuses on the subject as a whole, taking into account cognitive, emotional and social dimensions (49). Taking account of this ‘whole’, including the ‘imagined’ side identified in the girls we met, is also inspired by Edgar Morin’s reflections on the systemic approach and complexity (50, 51).

Given this gendered ‘social obligation’, this development can be seen as a strategy of self-assertion ‘under social influence’. In order to stand out from the crowd, to show that they are capable of considering these pictorial dimensions, this form of symbolism in the way they represent the world is a way of asserting their uniqueness within a social whole. These mechanisms are developed at an early age and are rooted in development, which is essential for understanding behavior in adulthood. These mechanisms can be seen as a form of protection against societal pressure, helping to explain women’s tendency to take things one step at a time and to take more time than men. This reading of the data is congruent with the international literature on social roles and gender distinctions (46–48, 52, 53).

Along the same lines, it is also interesting to compare our results with those from other international publications on children’s conceptions of health (25) to better understand behaviors in adulthood. In some studies listed in a literature review (25), the theme of diet is very present in the perceptions of children who attribute to it a central role in being healthy (29, 54, 55). These results are interesting to qualify according to gender, which is the focus of the study presented here. While diet is considered a major determinant for girls to be healthy and protect themselves from cancer, boys mention it less, regardless of age group. These perception results echo the behaviors of men and women in adulthood. Women are more inclined toward healthy eating, preferring fruits and vegetables to meat and alcohol (56, 57). These results show that women tend to take better care of themselves and pay more attention to their health than men, particularly by obtaining more information (media, discussions with health professionals, etc.) (56). Practices in seeking care reveal different behaviors depending on gender. Women consult earlier and more frequently for preventive measures, while men go to the doctor more often for health problems (58). These results are consistent with the perceptions of the young girls we met aged 6 to 18, who attach more importance to prevention than young boys.

Furthermore, physical activity is a dimension for which gender is a major determinant: very little discussed by girls, very present in the discourse of boys. It is interesting to compare these results with those in the literature to highlight the levels of practice and understand these differences. We observe a greater decrease in daily physical activity among girls than among boys (59, 60). This is explained by the influence of social norms (45), peers, family and by the transformation of the body (61). For many researchers, the differences in practices between girls and boys are not linked to disparate levels of natural abilities but would rather be the consequence of differentiated socialization between the two sexes: girls and boys, women and men adopt behaviors corresponding to gender roles and stereotypes (62–64). This form of normativity echoes what is called social representation. It is “socially developed and shared knowledge” (65) whose objective is to master and understand our environment and the events that occur in order to act and answer the questions we ask ourselves. But this vision does not allow for taking into account singularity and confines the subject “in a social whole” (5). The risk is to no longer recognize the singular capacity of any subject to construct meaning since it would be “an image of collective reality strongly suggested by society to the individual” (5). Taking an interest in people’s conceptions and perceptions thus allows us to question the place given to the singularity of the subject (4, 7, 66). Addressing what determines health cannot be done without identifying the positioning of children and adolescents on this subject and without taking gender into account. In order to prioritize health interventions, prevention and especially the chosen inputs can also become more effective. The results obtained through a multi-phase methodology demonstrated the relevance of taking the time to focus on this type of data (27).

The e.Photoexpression© has made it possible to highlight trends and predominances of discourse according to gender. In total, girls aged 6 to 18 are significantly more focused on 19 determinants while boys’ discourse is grouped around 3 determinants (28).

From a qualitative and quantitative point of view, the discourse becomes denser and broader during the Photonarration which offers a better understanding of how conceptions are organized systemically, that is to say how they combine and influence each other reciprocally. This phase highlights the fact that girls are in a more advanced form of argumentation than boys (Figures 8–14). With these strategies for pictorially representing the results, it is then possible to identify the complexity of the relationships that girls and boys establish or do not establish between their conceptions. By mobilizing them in this way, these health conception systems guide the argumentation and analysis of a situation in order to position oneself by referring to one or more conceptions to find points of stability or consider solutions. This constitutes a rationality specific to each child and which makes their argumentative specificity (27). We see that this process is more developed in girls than in boys. It is therefore appropriate to start from what they mobilize to enrich the argumentative spectrum of boys and help them mobilize more dimensions to find solutions to the life situations they will face. This approach echoes the work of Vygotski (67) on the proximal zone of development. For this author, this is the area of difficult learning that becomes accessible to the subject through support. “What the child is able to do today in collaboration, he will be able to do alone tomorrow” (31). It is also important to keep in mind that in children, psychoaffective and cognitive development is a complex process that leads them to question their surroundings and themselves (68). This process is not linear and does not boil down to constructing definitive answers, particularly with regard to the questions they ask themselves in terms of health. These are part of their openness to the world, which gradually becomes wider and is accompanied by a strengthening of their socialization, which is essential for the development of their autonomy (31). These reflections potentially constitute a decision-making aid for prevention support in order to strengthen the level of knowledge of children and adolescents in the dimensions that they do not address or address little, and to increase their decision-making capacity in health. This issue is applicable, from childhood to adulthood, through the construction phases that take place in adolescence. Advancing age has revealed very interesting results on the differences between girls and boys: the discourse trends of girls and boys change and their level of argumentation as well. It becomes richer in girls and poorer in boys. These data are in line with the evolution of health behaviors of children and adolescents (59, 60) and reflect life trajectories. The concept of life trajectory illustrates the path corresponding to the different moments of self-construction (23): birth, childhood, adolescence and adulthood. In order to support these life trajectories, it would be interesting to think about prevention through a differentiated and gendered approach: how to support the systemic vision of girls and broaden that of boys? It would not be a question of separating them but of probably supporting them differently in order to combat inequities (69, 70), particularly those related to gender. If we want to address themes that are less present in girls’ discourse, we can rely on the way boys talk about them to try to raise girls’ awareness. Conversely, putting girls’ perceptions under pressure on a theme that boys have little idea of is an interesting way to arouse their interest. Adopting this approach could contribute to better information by taking advantage of what is called the “peer effect.” Working on prevention methods early on Kempf et al. (66) with an entry through contexts linked to the living environment, current events but also taking into account gender and life trajectories seems to be a promising approach and perspective.

And because there is a real link between health and learning and because schools are attended daily by all children and adolescents, it is up to the school to support them in passing through these stages of their life trajectory. This common base contributes to a dynamic of success: education contributes to maintaining health and health ensures the conditions necessary for learning (71). This is why National Education must be able to provide children and adolescents, throughout their schooling, with health education in conjunction with all teaching (72). This role of the school is all the more important since the knowledge acquired during childhood will influence behavior in adulthood (73).

## 5 Conclusion

This study therefore provides new data on the way in which children and adolescents, girls and boys, do or do not enter into an argumentative process and systems thinking to define what, in their opinion, determines favorably or unfavorably their state of health and the occurrence of cancer. These results show that societal action can influence and normalize the perceptions of girls and boys from a very early age. Indeed, the example of the greater reduction in daily physical activity among girls than boys (59, 60) and the results on the differences in gendered perceptions described above can be explained by the influence of social norms (45, 61). Above all, these results reveal the need to think differently about prevention. All the more so as physical activity and the fight against sedentary lifestyles are public health issues (74, 75). In this respect, we need to think differently about how we support young people in terms of health education and prevention, in order to limit social modelling and the standardization of discourse. The creation of safe sports facilities, particularly for women and girls, could be a first step in this direction. The presence of non-mixed changing rooms, toilets, showers and access to menstrual hygiene should be taken into consideration to encourage girls to take part in activities. This can also involve adapting the rules of certain sports. For example, in rugby, the touch rule would replace the tackle rule, which can sometimes be a barrier for girls who have been told for a long time that such a sport is not for them and most of whom never had the opportunity to play it when they were younger. Implementing this type of strategy would also promote gender diversity in sport and therefore female participation.

However, the explanatory hypotheses for the trends identified by gender cannot be limited to social factors as a single interpretation. The information gathered shows that the life trajectories, the level of mastery of language and its symbolic value are revealed in part by the particular ways in which girls and boys express themselves. It would therefore seem important to be able to establish a preliminary diagnosis before each intervention in order to adapt and be as close as possible to the reality and needs of the target audience. Identifying the different profiles of the participants and spotting trends in their discourse would save precious time in analysing the group’s needs. The challenge in terms of prevention and reducing social inequalities in health would be to know how to manage and control the differences between the collective and the individual, between the most fragile profiles and those who argue the most in order to get to grips with the subjectivity of the child or teenager (69, 70). The discourse should therefore not be identical for all, with a normative character. It should take account of specificities and singularities so as not to clash with the context in which the young person finds himself. The opposite would result in the discourse being rejected, especially if it is too far removed from everyday reality. It also seems important to find leverage points and entry points that make sense for the child or adolescent, while offering tools to support them and give them the power to act as part of an empowerment initiative (80). Empowerment aims to develop skills to strengthen autonomy and the ability to act. Building these capacities seems to be an effective means of action, especially as the perceptions described in the results of this study will have a definite impact on life trajectories at different stages of life (73). This is why health education and prevention should be part of a life-course corresponding to the different stages in the construction of the self. As they move through these stages, their aim should be to help each child and adolescent gradually acquire the resources they need to make decisions that are good for their health, by adopting behaviors that will lead them to make their own decisions. To achieve this objective, it is essential to take account of children’s and teenagers’ perceptions of the determinants of health and cancer, by incorporating gender-and age-related factors into prevention strategies to establish anchor points that are close to the current and future needs of these populations. This information is crucial in helping professionals to work more closely with young people, so as to intrinsically stimulate their desire to learn, which is already present, but also to determine the priorities for intervention. The results of this research provide a set of indicators on which to base decisions.

## Data Availability

The original contributions presented in the study are included in the article/supplementary material, further inquiries can be directed to the corresponding author.

## Footnotes

^1^A school classified as REP has small classes and receives funding for projects, outings and interdisciplinary work.

^2^ https://www.pipsa.be/outils/detail-2139614080/e-photo-expression.html
